# Outcomes in the Older Appalachian Trauma Population After Falls Caused by Dogs: Who Let the Dogs Out?

**DOI:** 10.7759/cureus.110121

**Published:** 2026-06-02

**Authors:** Aliya G Burns, Elizabeth Mannino, Matthew Leonard, Hannah Collins, Bracken Burns

**Affiliations:** 1 Surgery, East Tennessee State University Quillen College of Medicine, Johnson City, USA; 2 Trauma Surgery, East Tennessee State University Quillen College of Medicine, Johnson City, USA; 3 Trauma, Ballad Health, Johnson City, USA

**Keywords:** accidental falls, dog ownership, fall prevention, fall-related trauma, rural areas

## Abstract

Introduction

Falls continue to be among the leading causes of nonfatal injuries in the United States. Falls are common among the older population, and the Appalachian region is considered one with an aging population. Pet ownership is common and has even increased since 2020. Few studies exist on the topic of falls caused by dogs.

Methods

Data were retrospectively pulled from the trauma registry at a rural Appalachian Level 1 trauma center between January 1, 2017, and June 15, 2022. Patients ≥18 who presented to the facility due to falling or tripping caused by dogs were included. Injuries due to dog bites were excluded.

Results

A total of 94 patients were included in the study, all of whom sustained an injury. Patients ≥65 years represented 68.1% of the cohort population. Females represented most of the population with 73.4% (P<0.001).

Hip/pelvic fractures were the leading cause of injury and were a significant predictor of outcomes for the older population. Hip fractures were significantly higher in those aged ≥65 years compared to those <65 years of age (92% vs 7%, P<0.001). The ≥65-year group also had significantly longer hospital length of stay (4.5 vs 3.2 days, P=0.046). Patients aged ≥65 years were 10.3 times more likely to be discharged to a care facility and were discharged most often to skilled nursing (odds ratio (OR)=10.292, 95% confidence interval (CI)=3.194-33.161, P<0.001).

Conclusion

Females were more likely to experience trauma because of a dog-related trip or fall. Older people are more prone to falling due to dogs and experiencing hip/pelvic fractures. These patients are also more likely to be discharged to a care facility, most commonly skilled nursing.

## Introduction

Unintentional falls were the leading cause of nonfatal injury in the United States in 2023 [1]. Notably, unintentional falls were also the leading cause of deaths from injury in individuals aged ≥65 years, with nearly one in four adults ≥65 years reporting a fall in 2020 [2].

In areas with a growing elderly population, such as Appalachia, a geographic region of the eastern United States including portions of several states that contain the southern Appalachian mountains, addressing falls becomes an even greater challenge. In 2016, individuals aged ≥65 years made up approximately 17.6% of the population in Appalachia, higher than the national average of 15.2% [3]. The rural Appalachian elderly population is especially vulnerable to falls and fall-related injuries due to the limited healthcare facilities and potentially long transport times to hospitals.

Although many factors can play into fall risk, one of the less-studied mechanisms is dogs. In 2024, 45.5% of households in the United States owned at least one dog [4]. Health benefits from dog ownership have been extensively researched. Dogs have been shown to decrease cardiovascular risk [5], increase physical activity [6], and reduce feelings of loneliness in their owners [7].

Unfortunately, dogs can also be a significant source of injury. Though much of the research to date has focused on dog bites, dogs represent an underrecognized cause of falls. One study used data from a national database to determine that females are 2.1 times more likely to experience a dog-related fall than males, and injury rates are the highest among individuals aged ≥75 years [8]. The most common injuries reported were fractures, contusions, and abrasions, predominantly affecting the extremities [8]. These findings are particularly relevant in the context of Appalachia, where an aging population, rising rates of dog ownership, and already elevated fall risk converge to create the conditions for a regional public health crisis. Investigating the epidemiology of dog-related falls in this population represents a timely and meaningful contribution to the broader literature on fall prevention.

To our knowledge, there is limited literature that evaluates dog-related fall outcomes. This study seeks to expand the literature on falls caused by dogs, especially in regard to rural populations, and aims to describe the demographics, injury patterns, and outcomes of adult patients presenting to a rural Level 1 trauma center after falls caused by dogs and to compare these characteristics between patients aged <65 and ≥65 years. We hypothesize that older patients with dog-related falls present with more severe injuries and worse outcomes.

## Materials and methods

Study design

This is an Institutional Review Board (IRB)-approved, retrospective database study at a rural Appalachian Level 1 trauma center.

Data collection

Data were abstracted from the trauma registry at a rural Appalachian Level 1 trauma center. The study period was between January 1, 2017, and June 15, 2022.

Inclusion and exclusion criteria

Patients aged ≥18 years who presented to the trauma center due to falling or tripping caused by a dog were included in the study population. Patients were identified using ICD-10 codes related to mechanisms caused by the presence of the dog. Patients <18 years, patients who had falls not related to ICD-10 codes that included dogs, and patients who experienced dog bites were excluded from the dataset. 

Statistical analysis

Data were obtained from the trauma registry at a rural Appalachian Level 1 Trauma Center. The registry platform was NTracs V5 (Digital Innovations; Austin, TX, USA). We looked at the patient population as one collective group and then divided them into two groups based on age. One group comprised patients ≥65 years, and the other comprised patients <65 years. Data abstracted included age, total hospital days, total intensive care unit (ICU) days, injury severity score (ISS) [9], sex, manner and location of injury, disposition, and location characteristics. Any falls that occurred on a porch were classified as outdoor. ISS was calculated within the registry software using the three highest abbreviated injury scale (AIS) [10] scores in the formula ISS = (AIS1)^2^ + (AIS2)^2^ + (AIS3)^2^. Descriptive statistics were used to observe age, total hospital days, total ICU days, ISS, sex, manner of injury, and location characteristics for the whole study population. The two age groups were analyzed using a t-test, looking at total hospital days, total ICU days, and ISS. Chi-square analysis was further used to analyze injury locations between the groups. Binary logistic regression was used to evaluate the disposition between the two groups. JASP (version 0.17.2.1; University of Amsterdam, Amsterdam, The Netherlands), an open-source statistical software package, was used to analyze the data [11]. P-value<0.05 was considered significant.

## Results

Ninety-four patients sustained injuries after a dog-related fall and were included in the study. Descriptive statistics revealed that there were 64 patients ≥65 years, representing 68.1% of the population. The average age of the entire population was 68 years. An average of four total hospital days, three ICU days, and an ISS of seven were seen in the overall study population (Table 1). 

**Table 1:** Descriptive statistics of 94 patients with injuries after dog-related falls using JASP v. 0.17.2.1 (University of Amsterdam, Amsterdam, The Netherlands) [11] IQR: interquartile range; SD: standard deviation; ICU: intensive care unit

	Mean (IQR)	Minimum/maximum	SD
Age	68.489 (18.75)	29/90	15.061
Hospital days	4.128 (3)	1/18	3.101
ICU days	2.909 (1)	1/11	2.773
Injury Severity Score	7 (5)	1/25	3.698

Descriptive statistics further revealed that almost three out of every four patients with dog-related falls were female (n=69, 73.4%). Walking or jogging with dogs was the activity that resulted in half of all falls (n=47, 50.0%). Tripping or pulling because of a dog was the highest known reason for falling (n=44, 46.8%). Most fall incidents occurred outside (n=54, 57.4%). Of the 94 patients with a dog-related fall, there were a total of 150 injuries. The most common injuries were hip/pelvic fractures (n=41, 27.3%). There were 27 extremity fractures (18.0%); two of these patients sustained both upper and lower extremity fractures. The third most common injury was to soft tissue (n=24, 16.0%).

Over half of the population were ≥65 years (n=64, 68.1%, P=0.049). T-test analysis revealed that this group experienced significantly longer hospital days (4.56 vs. 3.20, P=0.046). There was no significance between the ICU days (3.83 vs. 1.80, P=0.245) and ISS (7.33 vs. 6.30, P=0.211) (Table 2). They were also the only group to experience mortality, with two deaths occurring. 

**Table 2:** T-test analysis of patients 65+ versus <65 years that fell due to a dog using JASP v. 0.17.2.1 (University of Amsterdam, Amsterdam, The Netherlands) [11] IQR: interquartile range; ICU: intensive care unit

	65+, Mean (IQR) N=64	<65, Mean (IQR) N=30	t-statistic	P-value
Hospital days	4.56 (2.25)	3.20 (2)	3.38	0.046
ICU days	3.83 (0.75)	1.80 (0)	1.19	0.245
Injury Severity Score	7.33 (5)	6.30 (5)	1.28	0.211

**Figure 1:**
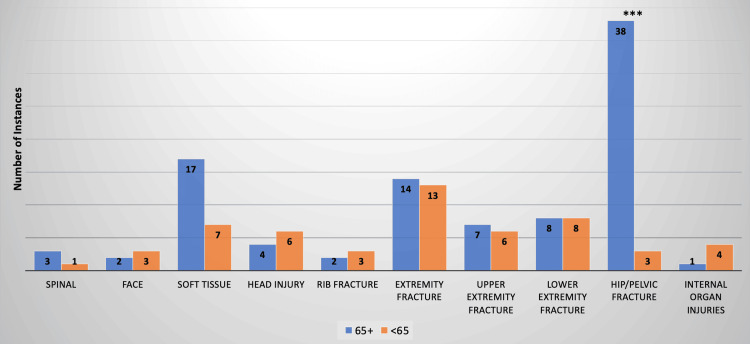
Chi-square analysis of injury counts between the age groups using JASP v. 0.17.2.1 JASP v. 0.17.2.1 (University of Amsterdam, Amsterdam, The Netherlands) [11]. *** P<0.001

Chi-square analysis showed that hip fractures were the only injury significantly higher in those aged ≥65 years (38/64 vs. 3/30, X^2^=20.28, P<0.001) (Figure 1).

Binary logistic regression showed that patients ≥65 years were 10.292 times more likely to be discharged to a care facility versus those <65 years of age (odds ratio (OR)=10.292, 95% confidence interval (CI)=3.194-33.161, P<0.001). These patients were also 4.697 times more likely to be discharged to a care facility if a hip fracture was present (OR=4.697, 95% CI=1.298-17.002, P=0.018).

## Discussion

Falls are the leading cause of nonfatal injury in the United States. Dogs, though a source of companionship and a catalyst for improved mental and physical health, can also increase an owner’s fall risk and are considered the cause of a rising injury burden [12]. While exercise is recognized as a key fall prevention strategy in older adults, and dog ownership can promote physical activity, clinicians should help patients weigh these benefits against the fall risk associated with dog ownership [13].

The results of our study reveal more severe injuries and worse outcomes in the ≥ 65-year-old population. This cohort had significantly longer hospital stays and significantly more hip/pelvic fractures than the younger patient group. Patients aged ≥65 years were also significantly more likely to be discharged to a care facility, representing a higher need for continued rehabilitation compared to younger patients. 

Hip fractures, the primary injury in patients aged ≥65 years in this study, tend to leave lasting effects. In a review article synthesizing data from studies with an average population age of ≥60 years, hip fractures were associated with significantly worse mobility, quality of life, and independence in function [14]. Only 40-60% of these patients regained their pre-injury mobility and ability to perform activities of daily living [14]. 

These results align with those reported in the literature. Other literature also recognizes that females and patients aged ≥65 years are more likely to present with dog-related fall injuries [8,15]. The most common injury type was musculoskeletal injury, which was also consistent with the literature. Our study revealed a disproportionate number of hip/pelvic fractures. Tripping or pulling was the highest known reason for falling, and walking or jogging was the most common activity associated with falls in our study, consistent with the literature [16,17].

Interestingly, this study noted that the majority of dog-fall injuries occurred outdoors, while previous studies have found that most occur indoors [8]. Appalachia’s rural region is home to many hiking trails and other opportunities for outdoor activities with dogs, which may explain this difference in injury location. It should be noted that this study was unable to determine whether dog ownership independently increases fall risk compared to other environmental hazards, as no control group of non-dog-related falls was included for comparison.

Limitations of this study included those of a retrospective, single-institution study. As a registry-based analysis, our cohort captures only patients who presented to this trauma center, which likely biases the sample toward more severe injuries. Additionally, the absence of baseline data in the registry on functional status, mobility, and pre-existing fall risk limits our ability to fully characterize the populations studied and isolate the independent contribution of dog ownership to fall risk. Additionally, the chi-square analysis of injury type was conducted at the injury count level rather than the patient level, which may violate the independence assumption. However, the primary finding of interest, the significantly higher rate of hip and pelvic fractures among patients aged ≥65 years, was corroborated by patient-level binary logistic regression, supporting the robustness of this conclusion despite the methodological limitation of the chi-square analysis. Finally, our registry data lacked the granularity to examine additional variables such as dog breed or size, terrain, and seasonal or weather-related influences on fall likelihood. Future research with more robust sample sizes should seek to address these limitations.

Fractures related to walking leashed dogs more than doubled from 2004 to 2017 [18]. This highlights the importance of developing injury prevention strategies that may reduce the burden of these injuries. Dog-related fall prevention strategies might focus on dog obedience training, especially given that half of the injuries occurred while walking or jogging with a dog. The need for ongoing fall prevention interventions in rural areas is recognized in the literature [19]. Trained dogs would be less likely to pull their owners over or walk in a way that causes the owner to trip. Dog walkers, especially those ≥65 years, might also be advised to avoid walking dogs on uneven or slippery surfaces. Finally, older individuals, particularly those with reduced mobility or pre-existing fall risk, may benefit from considering the physical demands of dog ownership before adoption in consultation with their multidisciplinary care team.

## Conclusions

Women and older adults represent disproportionately vulnerable populations for dog-related falls. Elderly patients were significantly more likely to sustain hip and pelvic fractures and be discharged to long-term care facilities. As the aging population and rates of dog ownership continue to grow, dog-related falls may represent an increasingly significant public health concern. Targeted injury prevention strategies tailored to older dog owners are needed, and future research should further investigate modifiable risk factors to better inform evidence-based interventions.
